# COVID-19: the implications for suicide in older adults

**DOI:** 10.1017/S1041610220000770

**Published:** 2020-04-30

**Authors:** Anne Pamela Frances Wand, Bao-Liang Zhong, Helen Fung Kum Chiu, Brian Draper, Diego De Leo

**Affiliations:** 1 Older Persons’ Mental Health Service, Concord Centre for Mental Health, Concord, Sydney, Australia; 2 Discipline of Psychiatry, School of Medicine, University of New South Wales, Sydney, Australia; 3 Affiliated Wuhan Mental Health Center, Tongji Medical College of Huazhong University of Science and Technology, Wuhan, China; 4 Chinese University of Hong Kong, Psychiatry Multicentre, G/F, TAi Po Hospital, Hong Kong, Hong Kong; 5 Australian Institute for Suicide Research and Prevention, Griffith University, Brisbane, Australia

Whether the coronavirus disease 2019 (COVID-19) pandemic influences suicide rates in older adults is not yet known. However, the pandemic is likely to result in a confluence of the risk factors for suicidal behaviors (Reger *et al.*, 2020) informing approaches to prevention. In this paper, we examine the links between suicide in older people and the COVID-19 pandemic, provide the perspectives of psychiatrists from four regions (China, Hong Kong, Italy, and Australia) facing different challenges and sociocultural contexts, and propose solutions to support older people.

## How COVID-19 may increase suicide in older adults

In an effort to reduce rates of infection, governments have adopted various policies such as social distancing, social isolation, and quarantine. Older people have been specifically advised to stay home given their vulnerability to COVID-19 and to reduce the burden on health services by limiting the spread of the illness. The adverse effects of isolation may be especially felt by older people (Armitage and Nellums, 2020) and people with preexisting mental illness (Druss, 2020). Living alone, loneliness, and social isolation are well-recognised risk factors for suicide in late life (Draper, 2014). Before the pandemic, even older adults living in senior housing communities designed to reduce social isolation described moderate levels of loneliness (Morlett Paredes *et al.*, 2020), presumably now exacerbated by quarantine and social isolation.

According to the interpersonal theory of suicide, suicide may be the result of thwarted belongingness and perceived burdensomeness, combined with an acquired capability for suicide (Joiner, 2005; Van Orden *et al.*, 2010). In a pandemic environment of social lockdown, older people may be especially vulnerable to suicide through a heightened sense of disconnectedness from society, physical distancing, and loss of usual social opportunities, as well as greater risk of anxiety and depression (Santini *et al.*, 2020). This may be compounded by feeling devalued or burdensome to society with the explicit knowledge that older people may not receive the health care they need due to resource rationing (Rosenbaum, 2020). During the pandemic, these perceptions may be heightened in older adults with depression and/or self-harm, who already feel a burden on society and their families (Crocker *et al.*, 2006; Wand *et al.*, 2018), and now hear from the media that they are deemed less worthy of care (Schwartz, 2020; Wenger and Schapiro, 2020). Such portrayals may contribute to suicidal behaviors in older adults by reinforcing negative internalized views on ageing as associated with loss of value and productivity and dependency (Crocker *et al.*, 2006; Wand *et al.*, 2018). Quarantine itself has been associated with psychological distress, especially when mandated (Brooks *et al.*, 2020). During the severe acute respiratory syndrome (SARS) outbreak, suicides were reported following enforced quarantine in a Taipei hospital (Barbisch *et al.*, 2015). Stressors associated with poor mental health outcomes and quarantine include longer durations of quarantine, frustration and boredom, insufficient information, inadequate supplies, and fear of infection, many of which disproportionally affect older people (Brooks *et al.*, 2020).

A key risk factor for suicide in older people is psychiatric illness, especially affective disorders (Troya *et al.*, 2019). The pandemic may result in new cases of affective disorders and create barriers to accessing treatment. During the SARS epidemic, high rates of psychological distress were associated with quarantine including symptoms of depression and post-traumatic stress disorder (PTSD), with greater prevalence of PTSD symptoms associated with longer periods of quarantine (Hawryluck *et al.*, 2004). Psychiatric disorders, PTSD more so than depression, may also be long-term sequelae of an epidemic (Mak *et al.*, 2009). Further, higher rates of probable PTSD and greater intensity of symptoms were found in residents of high SARS-prevalent areas compared to low SARS-prevalent areas, and in older people (aged 60+), even in those not infected (Lee *et al.*, 2006). The COVID-19 pandemic compounds this and other preexisting trauma in older adults, further contributing to risk of suicide and mental illness, and in addition to the “parallel epidemic” of anxiety, depression, and fear in the general community (Yao *et al.*, 2020).

The pandemic may also reduce access to psychiatric treatment. People with severe mental illness already experience discrimination and stigma, may be more susceptible to COVID-19 infection, have greater barriers to receiving timely medical care, and treatment may be less effective (Yao *et al.*, 2020). Those residing in nursing homes may be especially at risk of neglect with inadequate resources, overwhelmed staff (Thomas, 2020), and less community service in reach during lockdown. Regular appointments for mental health follow-up and prescriptions may be cancelled as determined to be “non-essential” or attendance hampered by disruptions to public transport, advice to stay home, and the media focus on emergency medical care, all undermining efforts to manage psychiatric illness in the community (Reger *et al.*, 2020). People presenting to Emergency Departments with suicidal behaviors may also be disadvantaged through overcrowding, long wait times (Reger *et al.*, 2020), and the prioritization of suspected COVID-19 cases and infection control measures, resulting in suboptimal care and follow-up and potentially influencing suicide rates.

Finally, it is widely expected that the COVID-19 pandemic will result in a global recession (Reger *et al.*, 2020), if not depression. The Great Recession in Europe and North America was estimated to have resulted in an additional 10,000 “economic suicides” between 2008 and 2010 (Reeves *et al.*, 2014), through mechanisms such as loss of employment, indebtedness, and housing insecurity (associated with depression, anxiety, and suicide). Financial insecurity for older people may be further exacerbated by the collapse in the stock market and low interest rates worldwide, reducing income from retirement savings (Reger *et al.*, 2020).

## International perspectives on the pandemic and suicide

China’s elderly suicide rate is relatively high, particularly for those living in central and rural regions (Zhong *et al.*, 2016). Wuhan, the largest city in central China with a population of over 10 million one-fifth of whom are aged 60+, was seriously hit by the COVID-19 pandemic. The lack of preparation for this sudden outbreak and mass quarantine measures adopted in all communities and villages of this city especially affected older adults. Initially, there were inadequate social support services for older people living alone. During the peak of the outbreak, some older adults could not receive timely and necessary medical services for their chronic diseases because routine services were cancelled in overwhelmed general hospitals, public transport was suspended, and concerns about acquiring the infection in hospitals. These barriers to treatment would be expected to increase distress and relapse of mental illness in older people (Yang *et al.*, 2020), increasing suicide risk. Later, community workers and volunteers were mobilized to provide social support services, groceries, and purchase medication for older residents. Due to the high case-fatality rate among infected older patients (Novel Coronavirus Pneumonia Emergency Response Epidemiology, 2020), older adults may have heightened fears of contracting infection and dying. Further, most older adults obtain information from television and radio, which relayed limited information about COVID-19 prevention and mental health care, resulting in anxiety and misinformation about the pandemic. Loneliness increased too, as Chinese older adults prefer face-to-face social interactions, interrupted by social distancing requirements.

The negative impact of all these factors on Chinese older adults may increase suicide risk. An online survey of 227 Wuhan-based older adults from 27 January to 2 February 2020 (B-LZ, unpublished data) revealed 39.2% had a low mood and 4.4% endorsed suicidal ideation in the last two weeks, clearly indicating need for urgent psychosocial and crisis intervention. Although there is no pre-pandemic comparison data for Wuhan, a recent meta-analysis revealed a 12-month prevalence of 9.7% for suicidal ideation in Chinese older adults (Wang *et al.*, 2020). The apparent lower prevalence in the Wuhan online survey is likely an underestimate given that it only assessed a 2-week period. Early on, China launched strategies for preventing and reducing mental health crises, including suicidal behaviors, in the general population (Li *et al.*, 2020). Guidelines and public health educational material for health professionals and the general public complemented new online mental health services and the positioning of mental health professionals in isolation hospitals (Li *et al.*, 2020).

The COVID-19 outbreak occurred at a time when Hong Kong was already devastated by the social unrest and economic downturn which started in June 2019 and continued until late January 2020, when the epidemic began. People were afraid to go out due to violent protests on the streets. The economy of Hong Kong had deteriorated sharply and many businesses closed. The prevalence of depression and post-traumatic stress increased substantially during the period of major social unrest in Hong Kong compared with previously (Ni *et al.*, 2020). Many health and community services for older people have been suspended or much scaled down since the start of the COVID-19 outbreak including mental health services, day hospitals, and daycare services. Core outpatient and inpatient mental health services have been maintained, but some older patients are afraid to attend hospitals for fear of contracting COVID-19, contributing to inadequately treated mental illness and associated suicide risk. In general, older people currently have heightened levels of depressed and anxious mood.

Hong Kong has experienced a severe epidemic before having been struck by SARS in 2003. In Hong Kong, SARS lasted just over 3 months, affected 1755 patients, caused ~300 deaths, and was associated with a sharp upturn in the elder suicide rate for 2003 (Chan *et al.*, 2006). The SARS epidemic was associated with increased risk of completed suicide in older women, but not men or the population aged under 65. Factors such as breakdown of social networks and limited access to health care may have been contributory. It was postulated that female elders, because of their preexisting ready engagement in social and health services, were more susceptible to the effects of temporary suspension of these services during the SARS outbreak (Chan *et al.*, 2006). The Hong Kong-specific Elderly Suicide Prevention Program established in 2002, with efficacy in reducing suicide rates (Chan *et al.*, 2011), has continued throughout the pandemic.

The traumatic experience of SARS, especially for the elderly, has predisposed to much fear and anxiety in older people in Hong Kong during the COVID-19 outbreak. This has been exacerbated by the lack of community and family support due to social distancing measures and reduced daycare services. The crisis and hardship to Hong Kong now are much more protracted than the SARS outbreak because of the preceding social unrest. While economic hardship and unemployment may predominantly affect younger people, the lack of medical, social, and community support particularly affects older people. It is likely that the suicide rates in both younger and older populations will increase in Hong Kong in the coming year.

In Italy, the COVID-19 epidemic developed with extreme virulence, disproportionately affecting older adults. According to the Istituto Superiore di Sanità (April 6, verified on a pool of 48,129 cases), the infection rates were 36% in people >70 years, with 83.6% of all deaths derived from this age group. Nursing homes paid an especially high price for the lack of protective measures and social distancing. In just 20 days, the nursing homes of Bergamo (Lombardy) had >600 deaths (Trabucchi and De Leo, 2020), a hecatomb. Combined, this has resulted in tragic television images of long lines of military trucks carrying coffins to incinerators, often very far from the deceased’s place of origin. There has been no way to celebrate funeral rites, nor accommodation in the cemeteries of the place of residence. The inability to accompany relatives in the last moments of life has been especially heartbreaking. The isolation imposed by the infection meant that thousands of people who subsequently died were last seen by family when they were taken to hospital by ambulance. The resulting widespread grief would be expected to increase suicide rates.

The pandemic is severely testing the entire Italian system, in particular welfare structures and a largely unprepared health service. The serious difficulties assisting the critically ill, combined with the scarcity of places suitable to receive such patients, and the insufficient number of ventilators have given rise to very painful ethical choices for health professionals on whom to provide care (Rosenbaum, 2020). This is widely reported in the media, likely heightening anxiety and distress in older people, who may choose suicide over uncertain health care.

During this period, national media (such as Corriere della Sera and Gazzettino) have reported at least five cases of suicide manifestly related to COVID-19. There are no reported cases of suicide among older adults, but there is strong concern for their physical and mental health (intolerance of being too long confined at home and often distressed by living with relatives or other virus-positive persons). There have been numerous calls for psychological assistance to helplines recently activated to meet the psychological needs of the people.

In Australia, the pandemic began not long after a disastrous bushfire season that has already had a huge impact in regional parts of Australia with over 3000 homes destroyed and an economic cost of >US$60 billion (Read and Denniss, 2020; Richards *et al.*, 2020). Before the pandemic, Australians were being encouraged to travel to tourist areas affected by bushfires in order to help with their recovery. Now travel is banned apart from that required for essential needs such as health care, groceries, work, or exercise. People aged over 70 and those with chronic conditions are advised to self-isolate at home.

For those older people living at home with the support of home care services, a new online training program is available for carers about infection control along with general advice about how to approach caregiving in the pandemic (Department of Health, 2020). There is an expectation that as the pandemic worsens that the carers will need to wear personal protective equipment to minimize cross contamination. This may be difficult for some older people to tolerate and could cause misunderstandings and distress particularly in those with cognitive impairment. There are reports that older and disabled people are cancelling home care services to minimize face-to-face contacts out of fear of contracting the virus (Uibu, 2020), with adverse implications for physical health care, another risk factor for suicide (Fassberg *et al.*, 2016). Family carers are being advised to minimize face-to-face contact with their older relatives. Grandparenting roles have been affected too with restricted contact for those living apart.

Suicide prevention organizations in Australia have recognized the “perfect storm” created by COVID-19 and are focusing their efforts. Lifeline, a charity which provides crisis support and suicide prevention, is focusing on mental health and well-being during COVID-19 through telephone lines, text, and webchat. RUOK is a non-profit suicide prevention organization which is also promoting connectivity during the pandemic through initiatives such as “connection cards,” which can be left on doorsteps with contact details for volunteers willing to listen and talk or provide practical support.

## Solutions: suicide prevention for older people during COVID-19

### Population approaches (primary prevention)

Various organizations have issued advice for coping with anxiety and stress during the COVID-19 pandemic, which may reduce suicide risk. The Australian Psychological Society (APS) has issued tips for older adults to stay mentally healthy (Australian Psychological Society Psychology and Ageing Interest Group Committee, 2020). Information is important to mitigate the risk of psychological distress, including providing older people with a clear rationale for why self-isolation is important, general education about the virus to reduce stigmatization, and emphasizing the altruistic decision to stay home (Brooks *et al.*, 2020). Conveying such information via television may be an effective approach that reaches many older people. The APS includes seeking information from reliable sources and in moderation, keeping concerns in perspective, and utilizing existing healthy coping skills. There are suggestions for coping with social distancing such as using videoconferencing, text messaging, phone calls, and e-mail with friends and family instead of face-to-face meetings. A sense of belonging, connectedness, and social support can be derived through online technologies (Armitage and Nellums, 2020), addressing key suicide risk factors. For some older people, this will involve learning new skills; Australians aged 65+, for example, are the most digitally excluded population group, least able to use digital technology for social connectivity and accessing information and services (Thomas *et al.*, 2017).

### Continuity of access to mental health care (secondary and tertiary prevention)

Community older persons’ mental health services should review their patient lists and screen for (Reger *et al.*, 2020) or otherwise identify clients who are especially vulnerable to mental illness and suicide (e.g. those who live alone, were already socially isolated, have chronic medical comorbidities or functional disability, are currently unwell, or who are at risk of relapse) and institute regular welfare checks and enhanced follow-up. Patients receiving depot antipsychotics or requiring regular medication monitoring (e.g. blood tests for lithium or clozapine) may need additional support from mental health services to ensure continued access to treatment, especially for those whose general practitioners have reduced hours or closed and no longer provide this care. People who contract COVID-19 and have suicide risk factors should be actively followed-up (Reger *et al.*, 2020).

Telehealth has rapidly come to the fore during the COVID-19 pandemic and may improve the access of older people internationally to mental health care now, but also later if embedded in mainstream health care. This involves switching from a hospital and clinic-based model of mental health care to telephone- and internet-based services and increasing public awareness about where and how to access the off-site services. There are online psychotherapy courses available for anxiety and depressive illnesses (see, e.g. https://thiswayup.org.au/) and for loneliness (Kall *et al.*, 2020), although specific telehealth treatments for suicidal ideation are less developed (Reger *et al.*, 2020). Grief counseling for those bereaved during the pandemic could also be delivered online. Older people may require additional support and education from families, friends, and healthcare professionals to access these services.

### Targeting loneliness and disconnection

Informal and professional services have a role in reducing social isolation – a factor increasing suicide risk – in older people during the COVID-19 pandemic. Grassroots initiatives to reduce loneliness have emerged such as #TheKindnessPandemic, developed by Celebrate Ageing, to promote acts of intergenerational kindness. Children can be encouraged to keep contact with their older parents to reduce fear and loneliness. For those without relatives, support services could be provided by community workers. Online technologies can also be utilized to promote a sense of belonging and provide social support for older people (Newman and Zainal, 2020). Formal services, such as Tele-Help/Tele-Check in Italy, telephone support, and monitoring, have demonstrated benefits in suicide prevention for older people (De Leo *et al.*, 2002). This model of proactive connection of older adults with health services via phone could be used to provide home assistance to older people at risk of suicide through social isolation, and/or psychological or physical illness. Variations of this approach could be delivered by family and friends, charities, voluntary organizations, or healthcare professionals (Armitage and Nellums, 2020). Telephone crisis line services too have played a role in suicide prevention and crisis support in the community providing an inexpensive, convenient and anonymous means of seeking support (Krysinska and De Leo, 2007) with the potential to reach a large proportion of community-dwelling older people (Chan *et al*., 2018).

### Mitigating the adverse effects of quarantine

Although quarantine is necessary to reduce the spread of COVID-19, measures can be taken to reduce the factors associated with poor mental health (Brooks *et al.*, 2020). This could include a public health campaign explaining why quarantine is important; minimizing the total duration of the quarantine period; ensuring older people have access to sufficient food, household essentials, and medicine (including through welfare strategies such as those implemented in China); and promoting suggestions for home-based activities to stave off boredom.

There may be unintended adverse consequences of policies to prevent contagion for older people. Advice regarding exercise and movement outside the home varies between countries and age groups, but social distancing may lead to more physical deconditioning, greater pain, and ultimately greater disability for older people. Each of these negative sequelae is also risk factors for suicide in older people (Fassberg *et al.*, 2016). Exercise is also an effective treatment for depression (López-Torres Hidalgo *et al.*, 2019), and if no longer available as a coping strategy for older adults, could potentially increase suicide risk.

## Conclusion

There are several ways in which the COVID-19 pandemic will have an impact on suicide in older adults, including by increasing the prevalence of known risk factors for suicide and infection control measures which increase isolation and vulnerability. Countries grapple with the pandemic crisis in the midst of their own challenges – economic, political, and natural disasters. However, there are common elements to suicide prevention in older adults: accessible dissemination of accurate information, promoting self-help and positive coping, reducing isolation through technology, and developing telehealth.
